# On Spinal and Spino-Gangleal Irritation

**Published:** 1831

**Authors:** 


### ON SPINAL AND SPINO-GANGLEAL IRRITATION. By W. R. WHATTON, Esq. of Manchester.

THIS is a practical dissertation, of considerable length and much merit, published in our North of England contemporary, on disease which has lately attracted a good deal of notice, but not more than it deserves. The author of this paper says, that the authority of Mr. Pott's name and opinions has, unfortunately, put a stop to inquiry respecting diseases of the spine—most surgeons after his time concluding at once, that all forms of spinal disease sprung from the same strumous source—caries of the vertebræ. Hence, he thinks, our diagnosis has been attended with confusion—our plans of treatment sometimes useless, sometimes even injurious. "No person, for instance, in these days, thinks of curing lateral curvature through the medium of caustic issues, while, on the other hand, caries is but seldom treated without them." After making a quotation from Copeland, where that surgeon regards pain on pressure, or increased sensibility to external heat, as indicative of spinal disease, Mr. Whatton observes as follows:—

"In the early stages of caries, as well as of lateral curvature, acute pain on pressure of the spinous processes of the vertebræ is rare, and when any great degree of increased sensation does exist in these parts, during the progress of the former disease, it does not occur until the bodies of the vertebræ are considerably affected, until the curve is formed, or until the processes have partaken, in common with other neighbouring parts, in the consequences of the disorder.

In cases of Spinal Irritation, on the contrary, this symptom is always one of the first and most constant, and it was in such cases, I apprehend, that the application of heat produced the severe uneasiness spoken of by Mr. Copeland. That gentleman constantly made use of this expedient, afterwards, for the detection of the early symptoms in cases of diseased Spine indiscriminately, and hence it followed, 'that he was unable to reduce the result to any given rules; and that sometimes he expected a great degree of pain, and it did not occur, while at other times, it took place where he least expected it.'

In those diseases commonly termed psoas abscess, also, whether arising from affection of the ligaments of the Spine, or of the intervertebral fibro-cartilages, or from caries of the bodies of the vertebræ themselves, there is scarcely ever any acute pain referable to the spinous processes of the back; and the symptoms are not unfrequently so very equivocal, that the true nature of the complaint is often overlooked or mistaken.

In many cases of caries, indeed, the patients do not experience sufficient uneasiness, at the time of the setting-in of the complaint, to induce them to notice their situation, and instances have occurred to me, in which the usual symptoms had been so entirely unnoticed, that there was no suspicion of the complaint existing, previous to the actual discovery of the curvature. —Not so, however, in Irritation of the nerves of the Spine.

In the writings of Messrs. Baynton, Wilson, Harrison, Shaw, and Dods, I do not perceive any notice of this affection; and it is not improbable that, as these gentlemen wrote expressly on the lateral curvature, they did not think it necessary to direct their attention to the various diagnostic appearances which characterize Spinal Irritation, or that it had escaped their observation altogether as a separate form of disease, and had, perhaps, in some instances, even been taken for the earlier stages of distortion.

However this may be, it is certain that there does exist a wide difference between these two forms of disease, each originating in a separate and distinct tissue, assuming a distinct type, and being followed by different sequels.

In cases of Irritation, the most prominent and characteristic symptom is the highly painful sensation produced by pressure on the points of the vertebræ, in that division of the spine where the disease is supposed to reside; *and this symptom is never wanting*. Its occurrence is to be explained, I presume, by the supposition that the irritation has already extended itself along the posterior nervous twigs, supplying the processes and arches of the vertebræ, and the numerous muscles and ligaments attached to them;—and its early appearance is easily understood, when we recollect that these twigs are the first which are given off by the lateral nerves on either side, and arise immediately from the spot implicated in the inflammation. There is another remarkable difference to be noticed with regard to this form of disease of the Spine. While cases of caries occur indifferently in the Spines of either sex, those of Irritation, like lateral curvature, are found chiefly among females. Of several hundred cases, which I have had opportunities of examining, I have not seen more than half a dozen occurring in male patients."

Mr. W. is not aware that any particular method of education, any kind of study, or any position of the body, predisposes to this complaint. He has met with it as frequently in the middle and lower classes of life as among the fashionable classes—among married females, the mothers of families, as among single ladies—rarely among girls. The youngest patient was thirteen years of age, and the oldest fifty. He has never seen it prove fatal. We shall now proceed to the—

SYMPTOMATOLOGY.

"In slighter cases, the symptoms are mild and intermittent, and the patients are able to attend to their various avocations, without much pain or uneasiness: and it is only when attention has been excited, by inquiry into the nature of their complaint, that they become aware of the extent of the disorder.

Irregular shooting pains in the limbs, and in the integuments and muscles of the chest and abdomen; occasional headach and loss of appetite; tremblings, and obscure uneasiness over the shoulders and down the back; with a general debility and disinclination to exertion or exercise, are the signs by which this form of the disease has usually manifested itself.

In the more urgent cases, the uneasiness becomes fixed and constant; the tremors are alarming, and the severe darting and lancinating pains over the chest and abdomen, and through the limbs, are harrassing and intolerable. Sometimes the cases had become protracted in their duration, and the patients had suffered for months under the most aggravated forms of the complaint, without any suspicion having been excited as to its real nature and origin;—and, in other instances, mistaken views of the disease had subjected them to various kinds of treatment, totally unnecessary, and, generally, quite inadequate to the removal of the complaint. In all these cases, however, there was one symptom, which, as far as my own experience has gone, has never been absent;—and that is a tenderness, upon pressure, in some part or parts of the spinal column.

In the slight cases, this pain is not so urgent as to cause much distress, and the pressure can be borne without any great suffering or disturbance; in others, the tenderness and excitement are so great, that, in running the finger along the spine, the instant the irritable spot is arrived at, the patient starts from under the pressure, and a degree of anguish is occasioned, so exquisite and excruciating, as frequently to produce the most violent spasms, which either go off gradually in repeated faintings, or subside into periodical and less painful dartings along the nerves running from the part which has been subjected to examination.

The darting pains correspond, in a remarkable manner, with the origin of the irritated nerves, and are very frequently found to strike through the chest, and to produce an acute smarting over the ribs and sternum, and throughout the neighbouring parts, where those nerves distribute their ramifications, and supply energy and sensation.

Every part of the Spinal Cord is subject to the disease: sometimes it is observed to fix itself upon one portion, and sometimes on another; frequently it is found existing in different portions, and occasionally over the whole column.

When the upper cervical nerves are affected by the irritation, the seat of the pain is most usually found in the suboccipital and lateral region of the cranium; the muscles of the face and the integuments of the neck are also affected; and there is a considerable degree of stiffness and inability to move the head and jaws. I have now and then met with a case where the pain has extended from the back part of the head, in a direct line over the skull, to the forehead, indicating a diseased state of the nerves supplying the occipital and frontalis muscles. When the affection fixes itself in the lower cervical portion of the Spine, the disease is generally announced by severe darting pains and cramps in the course of the axillary and brachial nerves, and along the upper and fore-arm, and by burning sensations, and aching of the muscles enveloping the shoulder joint, and upper and lateral parts of the chest. The severity of the pain is sometimes observed to fall upon the fore parts of the thorax, and to extend itself to the breasts; the glands of which become very painful to the touch, and are sometimes indurated and enlarged. This painful state of the glands of the breasts occurs as frequently in married females as in single women, and I have not perceived any thing to indicate a suspicion that one class of females is more liable to its attacks than another.

In these affections of the upper extremities and chest, there is usually a preternatural degree of lassitude and debility, frequent sighing, tremblings, and nervous twitchings; and sometimes also the wrists and hands are benumbed, and do not admit of their usual facility of direction. When the first division of the dorsal nerves forms the seat of the malady, we have the same painful shootings along the course of the anterior branches supplying the intercostal muscles, and edges of the ribs and sternum, and the upper parts of the epigastrium; and great soreness and aching in the ramifications of the posterior branches which go to the integuments and muscles behind the chest.

In the lower division, there is great pain around the abdomen and over the stomach; a feeling of soreness and smarting along the ribs; tightness around the chest, with frequently a considerable degree of loss of sensation and energy in the intercostal muscles; —these latter symptoms, with consequent dyspnœa, and a burning sensation over the sternum, and at the point of the xyphoid cartilage are, I think, never absent in well-marked cases of irritation of the dorsal nerves.

Atony of the abdominal muscles, causing much uneasiness and difficulty in expelling the contents of the bladder and rectum, is a constant symptom; and irregular pains, and sometimes partial paralysis in the integuments covering the lateral parts of the belly and thighs.

In the lumbar nerves, we have severe aching in the region of the loins, soreness over the skin and muscles of the genital organs and upper part of the thighs; painful and spasmodic dartings along the crural nerves, and down to the ancles and feet, with trembling, unsteadiness, and loss of power, similar to what is observed in the upper extremities. And in affections of the sacral nerves, the sacro-spinales and glutei muscles are found to partake in the disease, and the parts in the neighbourhood of the perineum.

In some acute cases of Irritation of the roots of the Spinal Nerves, or in those that have become chronic, the disease is very frequently seen to extend itself through the medium of the communicating branches, to the ganglial system; and in addition, therefore, to those symptoms, which have just been enumerated, we have others, consisting chiefly of irregular and spasmodic action of the involuntary muscles, and of the perverted functions of those organs and viscera, which derive their nervous energy from the ganglia to which the irritation has been continued.

When the disease has been carried from the spinal nerves of the upper part of the neck, by the correspondent branches of communication, to the cervical ganglia, the chief additional symptoms are violent and stabbing headaches, painful throbbings of the carotid and temporal arteries, and a fixed and heavy pain at the base of the skull, sometimes extending itself by the mastoid process, under the angle of the jaws, to the fore part of the neck.

When the middle half and lower portion of these nerves are in a state of irritation, or when the disease extends itself throughout the whole of the cervical spine, as is sometimes the case, the inflammatory excitement is communicated in like manner to the cervical ganglia, and thence, downwards to the cardiac nerves and cardiac plexus.

From this division of the ganglial system are furnished nervous branches going to the heart and lungs, to the aorta and the large blood-vessels of the parts situated within the thorax, and others which supply the involuntary muscles of these parts with their nervous energy. The heart and great blood-vessels are affected by irregular and spasmodic action; and are subject to various morbid and highly painful sensations; there is frequently severe aching and distress in the act of inflating the lungs, and a remarkable sensation of paralytic depression in the attempt to expel the air. Any of these symptoms readily occur when the patient has been alarmed by a sudden or unexpected occurrence, or when she has been hurried by any little increase of exercise or mental application.

As the disease advances, these symptoms are more frequent, more strongly marked, and are less easily removed. The intervals of freedom from the complaint gradually contract, until at last the patient becomes so irritable, and suffers such severe and continual pain, that her spirits are worn down, and she becomes weary of existence.

Should the disease have arisen in the dorsal region of the spine, the same affection extends itself to that division of the ganglial system which gives off nerves to the organs and viscera of the abdomen. The solar plexus wholly, the semilunar ganglia singly, or the splanchnic nerves and thoracic ganglia, appear in these cases to be affected by the malady: all those parts, indeed, of the abdominal cavity in succession, which receive energy from the solar plexus, or its secondary ganglia, are more or less subjected to the irritation. The diaphragm, stomach, liver and spleen, and the large and small intestines, and kidneys, as their appropriate ganglia are affected, become, in their turn, or together, liable to the encroachments of the disease.

If the stomachic plexus be the seat of the disease, we have painful depression at the region of the præcordia, especially after taking food; tenderness on pressing the stomach; difficult and incomplete digestion, attended with flatulence and preternatural distention; a feeling of anxiety about the heart, periodical and violent palpitations, and vertigo. These impressions being carried, through the medium of the cerebro-spinal connexions, to the brain, are frequently productive of sudden and distressing terrors and alarms, and the patients are occasionally tortured by the fear of apoplexy or some other fatal disease, of which, however, there is no real or perceptible indication.

The secretions of the stomach are greatly perverted; the gastric fluid becomes sour and unfit for the perfect solution of food; and whenever an attempt is made by the patient to extricate the nauseous air, which is plentifully formed, large quantities of acid watery fluid are brought up, and temporary relief is obtained from the removal of the distention and acrimony.

The biliary secretion in all probability too undergoes similar changes; and although the liver, perhaps, may not be endowed with a degree of sensation equal to that of the other viscera of the abdomen, yet severe pain in that organ is occasionally detected accompanying affections of the secondary ganglia of the central plexus.

The large and small intestines, when the mesenteric plexuses form the seat of the disease, are visited with severe twistings, and painful distentions, most frequently extending over the region of the colon, and producing intolerable anguish and distress. The sensibility of these organs is also very often perverted, the filaments sent off by these plexuses to the intestines being exceedingly numerous and highly susceptible.

In an extension of the disease from the lumbar nerves to the lower division of the ganglial system, we have painful affections of the kidneys and uterus. The menstrual discharges are commonly interrupted, and generally profuse, especially when the complaint has been of some duration."

We could not abbreviate, without injury, the foregoing symptomatology, and have therefore given a long extract, in the author's own words. We shall next advert to the

### Treatment.

In common cases of spinal irritation the treatment is very simple. Abstraction of blood from the part where tenderness has been discovered, by leeches or cupping, generally affords relief; and this is to be repeated, at intervals of three or four days, if the pains should return, as often as may be judged necessary. When the more urgent uneasiness has subsided, a small blister on each side of the affected vertebræ, or a single large one, above or below them, will be found beneficial. These must be repeated from time to time. Blisters are generally applied to the spine too soon after leeching, by which irritation is increased rather than allayed.

"In recent and slight cases, a single bleeding, or the application of a blister, will frequently succeed in effectually removing the disease; and I have known several instances where the complaint had been misunderstood, and had existed many months, and even years, which have given way without difficulty, when the curative means were applied to the true seat of the irritation, instead of the nervous filaments, which are the seat only of the distant symptoms. Some simple aperient medicine may be given, with a view of restoring the proper functions of the stomach and bowels, if it should be required, but more than this does not appear necessary; the depletion and blisters almost always proving sufficient for the removal of the irritation, and the restoration of the healthy functions following as a matter of course, as soon as the part has been properly relieved.

In the Spino-ganglial Irritation the same means are to be had recourse to, and it is necessary to attend, during the progress of the cure, to the state of the irregular secretions. In some cases, where the patient suffers from severe cardialgia, and is troubled with acid and flatulent eructations, I have generally been in the habit of prescribing the carbonate of soda or potass pretty freely, along with some simple bitter infusion; and in others, where restlessness and feverish excitement are urgent, they have been allayed by the use of the liquor acetatis ammoniæ, and small doses of opium and the submuriate of mercury.

Where the fixed pains have entirely subsided, but where there yet remains a sufficient degree of uneasiness to disturb the comfort of the patient, I have had recourse, as advised by Mr. Teale, to the use of some stimulant embrocation, which is directed to be rubbed over the spine occasionally; such as the liniment: subcarb: ammoniæ, or camphorated oil with spirit of turpentine. These applications keeping up a degree of stimulus or moderate counter-irritation, have been productive of very good effects. The flesh-brush, mustard poultice, or warm fomentation, will likewise answer very well.

Any debility consequent on the necessary depletion will soon be remedied by a gradual return to improved diet; and if any loss of appetite remain, the sulphate of quinine, or some of the preparations of iron may be useful; except, however, in chronic cases, or in some debilitated constitutions, these will seldom be required, and unless due attention have previously been directed to the state of the spine, they are inefficient and useless."

Some cases are detailed in illustration of the precepts here laid down, but we do not deem it necessary to insert any of them in this place. We think the paper very creditable to the practical talent of Mr. Whatton.

---

